# Hydrogel-Encapsulated Heterogenous Mesoporous Resin Catalyst for In Situ Anti-Cancer Agent Production under Biological Conditions

**DOI:** 10.3390/biom12121796

**Published:** 2022-12-01

**Authors:** Mahboubeh Nabavinia, Baishali Kanjilal, Manoj Pandey, Subash Jonnalagadda, Robert Hesketh, Manuela Martins-Green, Iman Noshadi

**Affiliations:** 1 Department of Chemical Engineering, Rowan University, Glassboro, NJ 08029, USA; 2 Department of Bioengineering, University of California, Riverside, CA 92507, USA; 3 Department of Biomedical Sciences, Cooper Medical School of Rowan University, Glassboro, NJ 08103, USA; 4 Department of Chemistry and Biochemistry, Rowan University, Glassboro, NJ 08028, USA; 5 Department of Molecular, Cell and Systems Biology, University of California, Riverside, CA 92521, USA

**Keywords:** mesoporous catalyst, Suzuki cross coupling, anti cancer pro drug, onsite drug delivery

## Abstract

A heterogenous Palladium anchored Resorcinol-formaldehyde-hyperbranched PEI mesoporous catalyst, made by one-pot synthesis, was used successfully for in situ Suzuki-Miyaura cross coupling synthesis of anticancer prodrug PP-121 from iodoprazole and boronic ester precursors. The mesoporous catalyst with the non-cytotoxic precursors were tested in 2D in vitro model with excellent cytocompatibility and a strong suppression of PC3 cancer cell proliferation, underscored by 50% reduction in PC3 cells viability and 55% reduction in cell metabolism activity and an enhanced rate of early and late apoptosis in flow cytometry, that was induced only by successful in situ pro drug PP121 synthesis from the precursors. The 3D gelatin methacrylate hydrogel encapsulated in vitro cell models underscored the results with a 52% reduction in cell metabolism and underscored apoptosis of PC3 cells when the Pd anchored catalyst was combined with the precursors. In situ application of Suzuki-Miyaura cross coupling of non-cytotoxic precursors to cancer drug, along with their successful encapsulation in an injectable hydrogel could be applied for tumor point drug delivery strategies that can circumvent deleterious side effects and poor bioavailability chemotherapy routes with concomitant enhanced efficacy.

## 1. Introduction

Chemotherapy, a primary treatment for early and late stages of cancers, is often overrun by biological and metabolic barriers limiting its therapeutic accumulation at the cancer site. It instead induces harmful collateral necrosis of healthy cells, multi drug resistance and recurrent tumor growth [1]. Poor tumor specificity or bioavailability can be mitigated via anticancer prodrug delivery circumnavigating tumor heterogeneity and collateral damage to benign cells [2]. Cancer prodrugs, with significantly lower cellular toxicity can be catalytically metabolized into an active form, in situ, enhancing on site bioavailability, therapeutic ability and reducing side effects [3]. Many prodrugs can be activated by the tumor microenvironment, like thiols, reactive oxygen species, and acidity or triggered by enzymes in the tumor microenvironment like β-glucuronidase [4], carboxypeptidase [5], or cytochrome P450 [6]. These factors, lack transformational specificity, precluding reliable application [3]. Instead, using transition metal catalysts like ruthenium, palladium, copper, or gold in biorthogonal in situ bond cleavage-based in vivo prodrug transformations show superior efficiency, specificity and reliability [7,8]. Palladium (Pd(0)) has shown excellent catalytic ability and biocompatibility for in vivo prodrug activation of proteins like phosphothreonine lyase by decaging propargylcarbamate-protected lysine residues [9]. Other examples entail in vitro bioorthogonal catalytic synthesis of anti-cancer agent 5-fluorouracil from biologically inert precursor [10] or the development and bioorthogonal activation of palladium-labile prodrugs of gemcitabine [11]. Catalytic stability and efficacy of nanomaterial anchored Pd(0) heterogeneous catalysts in simulated biological conditions has made bioorthogonal chemistry a mainstay in current chemical biology research, spatially targeted, prodrug-based, site-specific cancer therapies [10].

In this study, we examine a heterogenous Pd-anchored Resorcinol-formaldehyde-hyperbranched PEI nanostructure (MR-Pd) for in vivo, in situ catalytic Suzuki-Miyaura cross-coupling of iodoprazole and boronic ester to the anti-cancer agent, PP121 and its concomitant efficacy in 2D and 3D encapsulated structures containing PC3 cancer cells [12]. The conversion uses a mesoporous, palladium anchored mesoporous polymeric catalyst, synthesized as per our previous work [13]. The hydrogel for cell encapsulation comprises of GelMA-Sodium alginate combination, into MR-Pd is dispersed alongwith the non-cytotoxic cancer pro-drug precursors. In this work we have showed the feasibility of killing malignant cells, in a 3D encapsulated model, via in situ production of anti-cancer agent using cross coupling, under biological conditions. This can pave the way for an injectable hydrogel as a vehicle for possible introduction of catalyst-precursor combination at the tumor site. While the hydrogel sets under in vivo conditions, the same conditions allow for cross coupling of the pro drug precursors at in vivo temperatures. The anti-cancer agent PP121, thus produced in situ can kill cancerous cells by apoptosis.

## 2. Experimental Section

### 2.1. Synthesis and Characterization of Mesoporous Catalyst

The chemistry of synthesis of the mesoporous resin catalyst (MR-Pd) is replicated from our earlier published work. All analysis techniques for catalyst characterization are as per the methodologies used in our previous published work [13]. Briefly, resorcinol, 98%, formaldehyde 37%, palladium acetate and PEI 99% (molecular weight 10,000 DA) were purchased from Alfa-Aesar and used without purification. The mesoporous resins (MPR) and the palladium catalyst anchored MPRs were synthesized in one-step by the reaction between resorcinol, formaldehyde and PEI in water or ethanol solution. In a typical run 1.5 g resorcinol was dissolved in 4.4 mL of distilled water with vigorous stirring in a water bath (40 °C) to obtain a clear solution, to which 5.6 mL of 1.96 or 3.93 *w/v*% aqueous or ethanolic PEI solution was added. This was kept in the water bath for 15 min and followed by the sequential addition of palladium acetate powder was added under stirring. Then, 2.1 mL of formaldehyde was added with vigorous stirring, after which the solution was poured in a Teflon hydrothermal autoclavechamber and baked for 12 h at 120 °C. The MR-Pd catalyst so formed was washed with DI water and dried under vacuum at room temperature.

### 2.2. Procedure for In Vitro Pro Drug Synthesis and Assessment of Efficacy

The PC3 cells used here were a gift from Dr. Pandey’s lab at Copper Medical School, Rowan University and they were cultured in RPMI-1640 contained 10% FBS and 1% penicillin/ streptomycin. Cells were seeded in 24 well plate and after one days 10 mg of sterile catalyst (300 to 500 μm) added to each well. The viability of PC3 cells, grown on MR-Pd surface was evaluated by a commercial Live/Dead viability kit (Invitrogen, Thermo Fisher Scientific, Eugene, Oregon, USA), according to manufacturer’s instructions. Cells were stained with 0.5 μL/mL of calcein AM and 2 μL/mL of ethidium homodimer-1 (EthD-1) in PBS for 5 min at 37 °C. Fluorescent image acquired at days 1, 3, 5, and 7 post-seeding using an AxioObserver Z1 inverted microscope (Zeiss, White Plains, NY, USA). Viable and dead cells, appearing green and red, respectively, were quantified using ImageJ software (software version 1.5.0). Cell viability was the ratio of live cells to total number of cells. For flow cytometry Muse^®^ Annexin V & Dead Cell Kit was used for quantitative analysis on the flow cytometer (Guava^®^ Muse^®^ Cell Analyzer, Luminex Corporation, Seattle, WA, USA). The concentrations and percentage of live/dead/apoptotic cells were calculated by MUSE software (version V1.9.0.2).

A Fisher Scientific Cell Scraper was used to detach cells. 20 μL each of cell suspension and Muse^®^ Annexin V were mixed and incubated in the dark at room temperature for 20 min. This was then run in the machine with Annexin V & Dead Cell assay. Each sample was measured three times. Presto blue assay was carried out in a 12-well plate with test cells in cultured medium. 500 μL Presto/Blue solution (10% in medium without serum) was added to each well and the plates were incubated at 37 °C for a specified time period. Fresh serum-free media served as blank. After incubation, 100 μL of Presto/Blue solution from each assay well was transferred to a new well in 96-well plate. Change in fluorescence of test reagent was measured in a new plate by fluorescence multi well plate reader (Excitation/Emission: 530/590 nm). The cellular encapsulation was carried out in a GelMA-Sodium alginate combination. Sodium alginate powder was dissolved in PBS and sterilized by 0.2 μm syringe filter. Lithium phenyl-2,4,6-trimethylbenzoylphosphinate (LAP) was used as photoinitiator. GelMA was prepared according to standard literature [13]. For cell encapsulation, GelMA, alginate, and LAP was mixed to desired concentration and then cell suspension (5 × 10^6^ cells/mL) added to it.

## 3. Results and Discussions

**Scheme 1:**
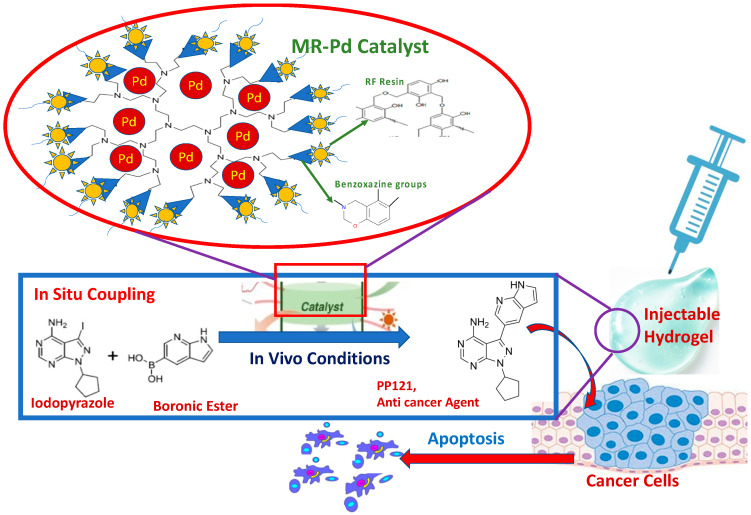
Mesoporous Resorcinol Formaldehyde-PEI catalyst anchors Pd(0) and non-cytotoxic cancer prodrug precursors. It is encapsulated in a hydrogel and set under in vivo conditions. The heterogenous catalyst catalyzes the in situ coupling of the non-cytotoxic precursors, under in vivo conditions to anti-cancer agent PP121, which kills cancerous cells by apoptosis.

Here, we anchor Pd(0) in a mesoporous polymeric resin network of hyperbranched polyethyleneimine (PEI) by a one-pot, template-free synthetic methodology, employed in our earlier published work [13]. Resorcinol and formaldehyde polymerize, react with PEI’s amine groups forming benzoxazine rings, complexing and anchoring the Pd atoms in a mesoporous polymer network. This is also depicted in Scheme 1 below.

The N_2_ adsorption, SEM (Supplementary Figure S1) confirm high surface area and porosity with a uniform microporous morphology and an interconnected network. The existence of Pd(0) is corroborated by XPS spectra while EDX elemental analysis confirm the presence of nitrogen atoms, from PEI, in the catalyst structure (Figure S1). We abbreviate the catalyst structure as MR-Pd. In our earlier work [13], we obtained a uniform dispersion of Pd nanoparticles without a template or cytotoxic organic solvents, paving way for scale up, low process toxicity and a better choice for biological applications. We also established good catalytic efficiency in mild conditions with Suzuki-Miyaura cross-coupling [13] achieving upto 98.98 ± 1% and 62 ± 5% conversion in batch and continuous microreactors, respectively. The chemistry tailorability makes its biological application possible via structural manipulation and/or dispersion in a carrier hydrogel.

For in situ, biological catalyst application for cancer treatment, the cytocompatibility of the MR-Pd catalyst structure was shown by live- dead fluorescence and Actin-DAPI staining assays recording negligible cytotoxicity (Supplementary Figure S2) [14]. This was corroborated by quantified cell viability and cellular expansion with the mesoporous catalyst comparable to growth media as control (Figure S2).

**Figure 1:**
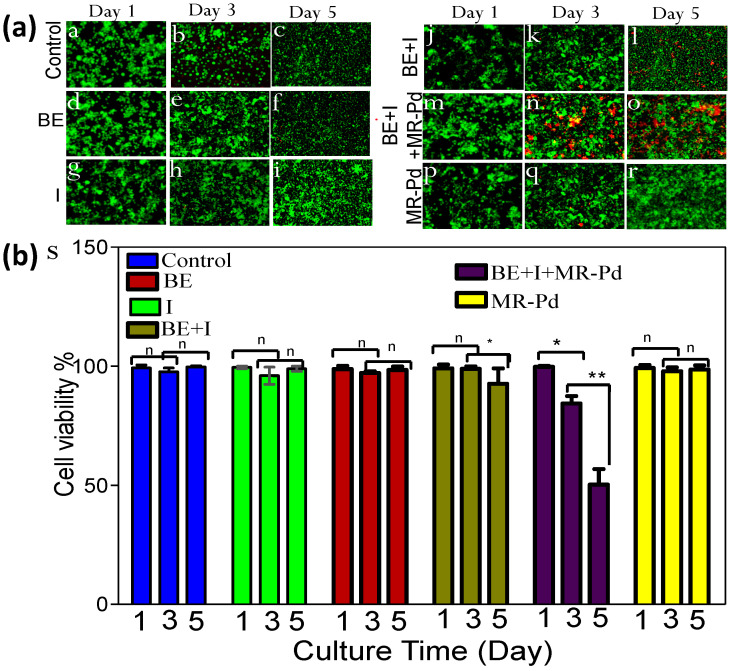
In situ drug synthesis for monolayer PC-3 cells culture model: (**a**) cell viability based on live & dead assay. (**b**) cell viability quantified [BE = Boronic Ester, I = Iodoprazole, MR-Pd = Mesoporous Catalyst]. “*” signifies *p* ≤ 0.05 (statistically significant), “**” signifies *p* ≤ 0.01 (highly statistically significant result).

This cytocompatible, MR-Pd catalyst was now tested for in situ drug synthesis in a standard monolayer culture, seeded with PC3 prostrate cancer cells for two days yielding a monolayer with 50% confluency. Live-dead cell viability assay (Figure 1a) showed an obvious increase in dead PC3 cells from 10% (day 3) to 57% (day 5) with a combination of the precursors (iodoprazole and boronic ester) and the MR-Pd catalyst [14]. This underscored possible MR-Pd catalyzed production of the PP-121 drug and its subsequent effect of killing PC3 cells. Quantitative cell viability (Figure 1b) corroborates this observation showing a significant drop in cell viability only when the precursors are combined with the catalyst underscoring that PC3 cells are probably killed only due to in situ drug formation.

Overall, the results confirm that PC3 cells death was induced only by successful in situ pro drug PP121 synthesis. The catalyst biocompatibility also means that the catalyst, on its own, cannot be the cause for PC3 cell death. The PEI in the MR-Pd catalyst structure orients the RF pre-polymer along the lines of its dendrimer structure, controlling the porosity type and extent. Porosity allows easy diffusion of the precursors, the high content of immobilized anchored Pd, evidenced in our earlier publication [13] promulgates their conversion into PP121, which then easily diffuses out mesoporous structure onto the cellular interface.

**Figure 2:**
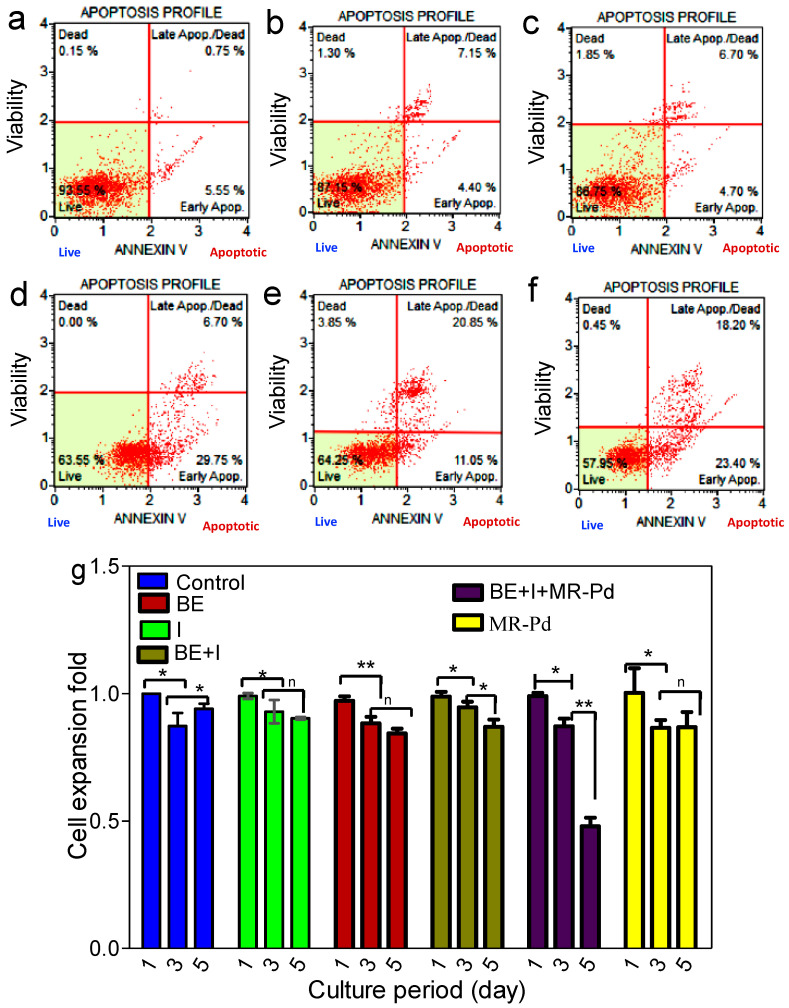
(**a**–**c**) In situ drug synthesis for monolayer PC-3 cells, Control, without catalyst:(**a**) Control day 1 (**b**) Control on day 3, (**c**) Control on day 5. (**d**–**f**). In situ drug synthesis in present of MR-Pd catalyst: (**d**) with catalyst, day 1 (**e**) with catalyst, day 3, (**f**) with catalyst, day 5 (Cell viability results based on flowcytometry assay). (**g**). Cell expansion fold based on presto blue assay [BE = Boronic Ester, I = Iodoprazole, MR-Pd = Mesoporous Catalyst]. Statstical ignificance showed using *p*-value on bar graph. “*” signifies *p* ≤ 0.05 (statistically significant), “**” signifies *p* ≤ 0.01 (highly statistically significant result).

In order to underscore the monolayer culture results, the mechanism of PC3 cancer cell death was studied using flow cytometry. Flow cytometry results (Figure 2a–f) showed an enhanced rate of early and late apoptosis, instead of necrosis [15].

The data point density shifts from the 3rd quadrant (live cells) to the 4th quadrant (early apoptosis) followed by the 1st quadrant (late apoptosis with Annexin V and Propidium Iodide fluorescence). There is very little necrosis for both control and test groups with few data points in the 2nd quadrant. The apoptosis rate, traversing via the 4th quadrant to the 1st appears significantly more rapid with the test set (Figure 2d–f) containing both the precursors (iodoprazole and boronic ester) and MR-Pd-catalyst, compared to control (Figure 2a–c) which had Precursors without MR-Pd catalyst. This proves that PP121 anti-cancer agent was produced in situ, only when the catalyst accompanied the precursors and that it was effective in killing PC3 cancer cells.

In addition, cell metabolism activity, measured by standard Presto Blue assay, also declined (Figure 2g) significantly only when the MR-Pd catalyst was introduced alongwith the precursors and not with any combination that left out either the catalyst or one of the precursors. The results, yet again, corroborated MR-Pd catalyzed cross coupling of iodopyrazole and boronic ester to PP-121 as the factor responsible for suppressing PC3 metabolic activity. The suppression of anaplastic thyroid carcinoma tumour growth by PP-121 has been reported to proceeds via mTOR-phosphatidylinositol-3-OH kinase inhibition and by binding to tyrosine kinases (VEGF receptor) [16].

While the 2D model underscored the catalytic cross coupling synthesis of PP121 and its mechanism on PC3 cell death, a 3D model is a more representative evaluation for drug screening since it better simulates tissue microenvironment [17,18,19]. The use of hydrogels as a support for nano or meso structures or encapsulating material for targeted injectables is a current topin of interest, having been explored in wide ranging applications, from tissue regeneration to drug delivery [20,21]. PC3 cells were encapsulated in GeLMA—alginate hydrogel to evaluate drug screening. The two compositions used to encapsulate PC3 cells were; G5AL: GeLMA (5% *w*/*v*)-Alginate (1.5% *w*/*v*)-LAP (0.1% *w*/*v*) and G7AL: GeLMA (7% *w*/*v*)-Alginate (1.5% *w*/*v*)-LAP (0.1% *w*/*v*). The encapsulant combinations were characterized for microscopic structure, swelling and degradation (Supplementary Figure S3a–c). With increased GelMA content, porosity reduced, degradation stability increased, while the swelling ratio was comparable. Encapsulated structures were tested by cell staining, cell expansion and the Western blot tests (Supplementary Figure S3e,f). The tests underscored the cytocompatibility of the encapsulant hydrogel matrix. The screening studies also proved the necessity of the MR-Pd catalyst’s presence in producing PP121 anticancer agent, in situ, from precursors. We carried out screening studies with and without the MR-Pd catalyst. We encapsulated the PC3 cells in the G7AL composition and started the in situ drug screening 5 days after encapsulation. Additionally, we encapsulated PC3 cells in G7AL with 5 mg MR-Pd catalyst, and started the drug screening test on day 3. In both set ups, the prodrug precursors were dissolved in same medium used in the monolayer study. The catalyst was added to the well plate or injected to the encapsulated cells at day 4 and evaluation commenced on day 5.

**Figure 3:**
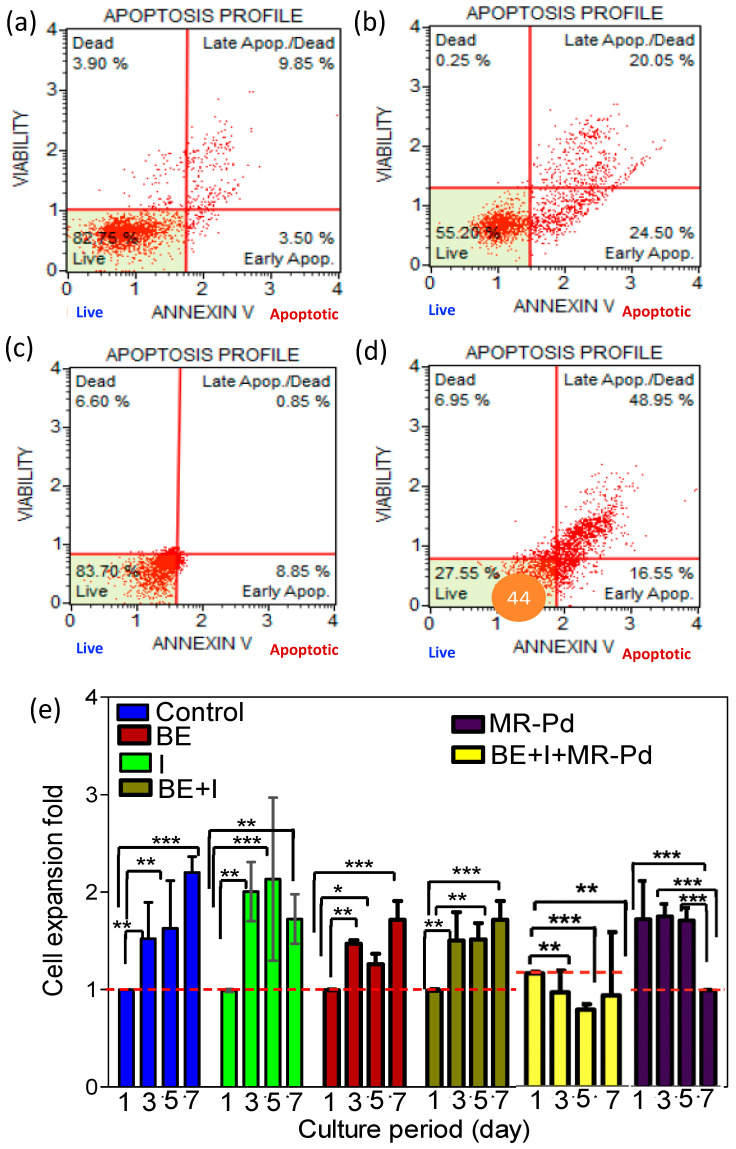
In situ drug synthesis for encapsulated PC-3 cells: (**a**,**b**) Flow Cytometry cell viability results on cncapsulated PC-3 cells in GelMA-Alginate without catalyst (7%:1% *w*/*v*) (**c**,**d**) Flow Cytometry cell viability results on cncapsulated PC-3 cells GelMA-Alginate with MR-Pd catalyst (7%:1%:0.1% *w*/*v*) (**e**) Cell proliferation based on presto blue assay. Encapsulated PC-3 cells in GelMA-Alginate in various combinations. [BE = Boronic Ester, I = Iodoprazole, MR-Pd = Mesoporous Catalyst]. Statstical ignificance showed using *p*-value on bar graph. “*” signifies *p* ≤ 0.05 (statistically significant), “**” signifies *p* ≤ 0.01 (highly statistically significant result), “***” signifies *p* ≤ 0.001(highly statistically significant result, null hypothesis can be rejected).

In this 3D model too, the cell death mechanism was via apoptosis rather than necrosis as evident in the flow cytometry results (Figure 3a–d).

The figures underscore that the encapsulant matrix or precursors (iodopyrazole and boronic ester) without the presence of MR-Pd catalyst have little to no effect in speeding apoptosis of cancer cells (Figure 3a,b). However, when the encapsulated cells are combined with the precursors alongwith the MR-Pd catalyst, there is a rapid scale up of late apoptosis of live PC3 cells. Negligible data points in the 2nd quadrant, prove that there is no necrotic effect of either catalyst or precursors or encapsulant matrix on its own. The cytocompatibility of the encapsulant matrix and of MR-Pd, explained above, are a testament to the results. The acceleration of cells from live to late apoptosis quadrant with the precursors-catalyst combination (Figure 3c,d) underscores in situ PP-121 production pushing apoptosis. Presto blue results showed in Figure 3e show a much stronger suppression of cell expansion in the encapsulated model containing precursors-catalyst combination. In no other combinations, that left out one or more of the components (precursor or catalyst), was suppression of cellular proliferation statistically significant.

The methodology employed in making of the MR-Pd catalyst used here, as per our earlier work, ensures mesoporous structure, high palladium complexing and uniform palladium distribution throughout the structure, ensuring steady conversion of precursors into PP121 [13]. We were able to encapsulate cells in a hydrogel, also containing the MR-Pd catalyst and underscore its successful application in a 3D model. Hence, a hydrogel based injectable patch can be envisaged and designed, to target tumors. The hydrogel can be loaded with the MR-Pd catalyst and precursors, which can harness the catalyst to generate steady bioavailability of the anti-cancer agent, produced under biological conditions. The transport of in situ generated PP121 agent out of the mesoporous catalyst structure in such a case could be guided by a concentration potential difference. The 3D encapsulated cellular model explored here establishes an outlook for drug screening by corroborating in situ PP-121 production under biological conditions and its efficacy in ensuring apoptosis of PC3 cells. Negligible necrosis is induced by any components. The model studied here opens up new vistas in possible on-site tumor alleviation. It can be scaled up for a practical injectable treatment modality, bypassing deleterious side effects of chemotherapy while proffering superior bioavailability, treatment efficacy at a reduced cost. In our earlier work, we also showed the application of these MR-Pd catalysts for Suzuki-Miyaura couplings in continuous microreactors, achieving conversions up to 62%. In the near future, it is possible to envisage smart applications that incorporate bio printed miniaturized, microchannel reactors with implantable pumps, made from packed beds of this MR-Pd catalysts, that can push in designated, and calculated amounts of PP121 cancer agent, produced in situ under biological conditions, bypassing cytotoxic effects on healthy neighboring tissues.

## 4. Conclusions

A Palladium anchored mesoporous polymeric resin heterogeneous catalyst (MR-Pd) was prepared and used successfully for the in situ Suzuki-Miyaura coupling synthesis of cancer pro drug PP-121 from precursors iodopyrazole and boronic ester. The cytocompatible MR-Pd catalyst showed excellent catalytic activity for, in situ, converting the precursors to anti-cancer agent PP121 under mild, biological conditions, killing PC3 cancer cells in both 2D and hydrogel encapsulated 3D models, without causing any collateral cytotoxic or necrotic damage to healthy cells. The pathway of cellular death, measured by flow cytometry, was via the apoptotic mechanism rather than necrosis. The successful in situ application of the Suzuki-Miyaura cross coupling reaction, which can work under mild in vivo conditions and a simulated biological environment offers a strategy to the development of injectable hydrogels to deliver the catalyst and prodrug agents directly to the cancer or tumor site without incurring the deleterious side effects and the poor bioavailability of chemotherapy routes while enhancing treatment efficacy with a concomitant reduction in treatment cost.

## Data Availability

Not applicable.

## Supplementary Materials

The following supporting information can be downloaded at: https://www.mdpi.com/article/10.3390/biom12121796/s1, Figure S1: Characterization of MR-Pd (a): Surface area of MR-pd computed from N2 adsorption (b): SEM image of MR-pd (c): EDS elemental mapping: Analogous elemental mapping of carbon, oxygen palladium and all other elements, scale bar 5 micrometer (d–f): XPS spectra of C1s, N1s and Pd3d (g): Area EDS spectrum for atomic and weight percentage of various elements; Figure S2: Biocompatibility of MR-Pd catalyst (a) Live & dead assay fluorescent microscopic images (b) Quantitative cell viability based on image processing (c) Actin-DAPI staining day 5 (d) Cell expansion fold based on presto blue assay; Figure S3: Evaluation of encapsulated prostate cells (PC-3) in hydrogel (a) SEM (b) swelling ratio (c) degradation percentage of various hydrogel composition (d) cell staining by live & dead assay (day 1) (e) cell expansion based on presto blue assay (f) Western Blot of encapsulated cells.
