# Supplementary material for: Hydrogel-Encapsulated Heterogenous Mesoporous Resin Catalyst for In Situ Anti-Cancer Agent Production under Biological Conditions
Source: Biomolecules. 2022 Dec 1;12(12):1796. doi: 10.3390/biom12121796 (PMC9776059; doi:10.3390/biom12121796)
Supplement: Supplementary file 1 [file biomolecules-12-01796-s001.zip › biomolecules-2006345-supplementary.pdf]

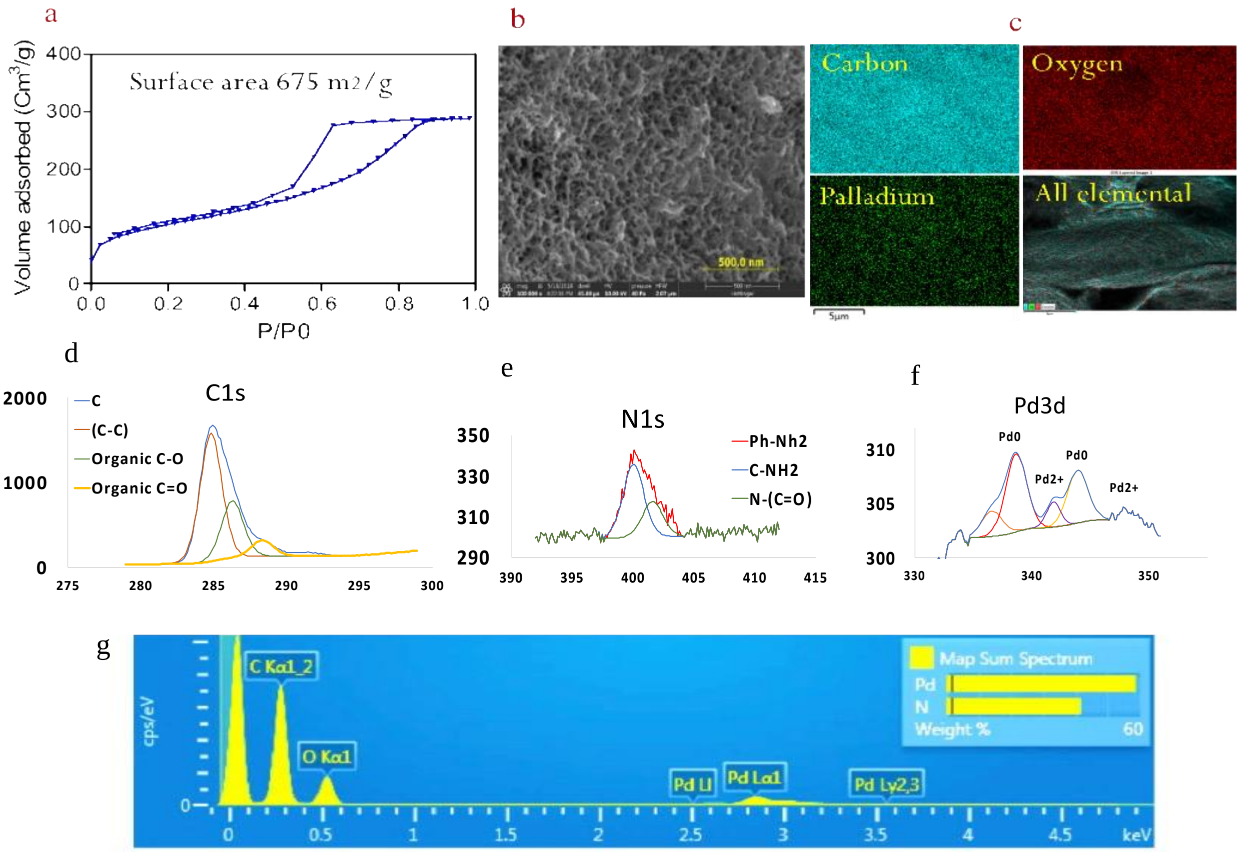

Figure S1: Characterization of MR-Pd (a) : Surface area of MR-pd computed from N2 adsorption (b): SEM image of MR-pd (c): EDS elemental mapping: Analogous elemental mapping of carbon, oxygen palladium and all other elements, scale bar 5 micrometer (d-f)XPS spectra of C1s, N1s and Pd3d (g): Area EDS spectrum for atomic and weight percentage of various elements [13]

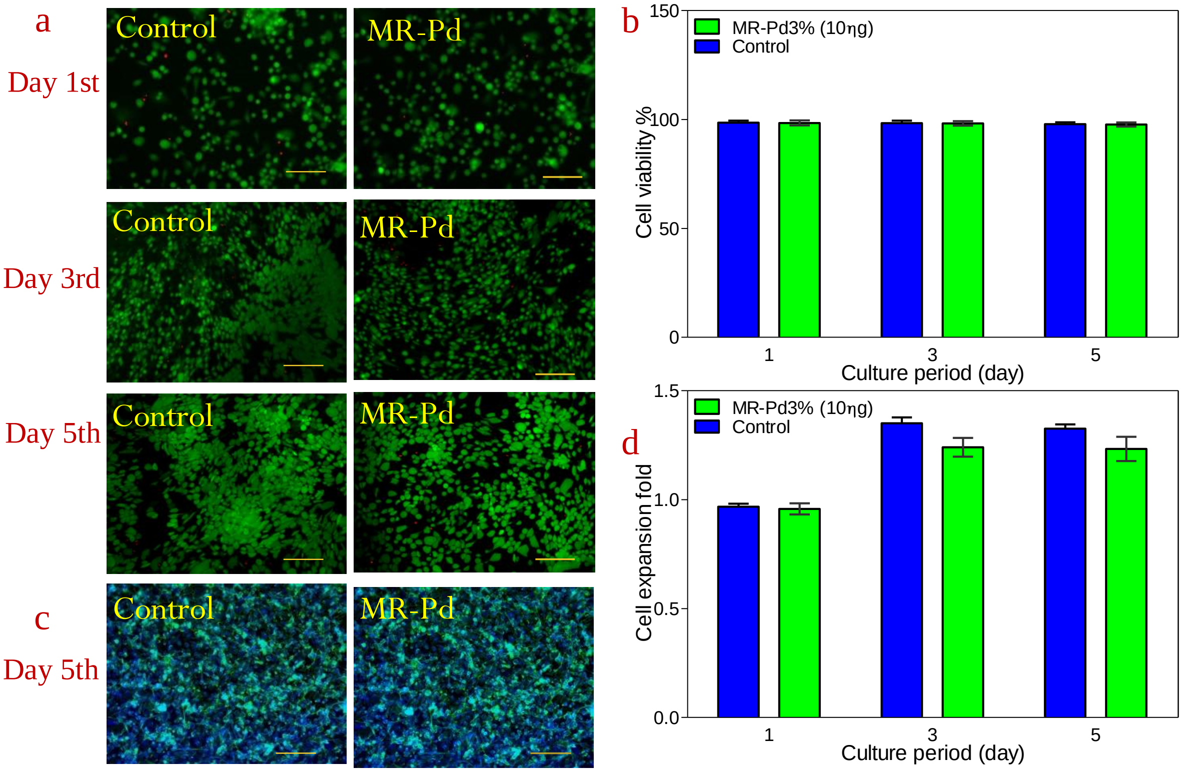

Figure S2: Biocompatibility of MR-Pd catalyst (a) Live & dead assay fluorescent microscopic images (b) Quantitative cell viability based on image processing (c) Actin-DAPI staining day 5 (d) Cell expansion fold based on presto blue assay

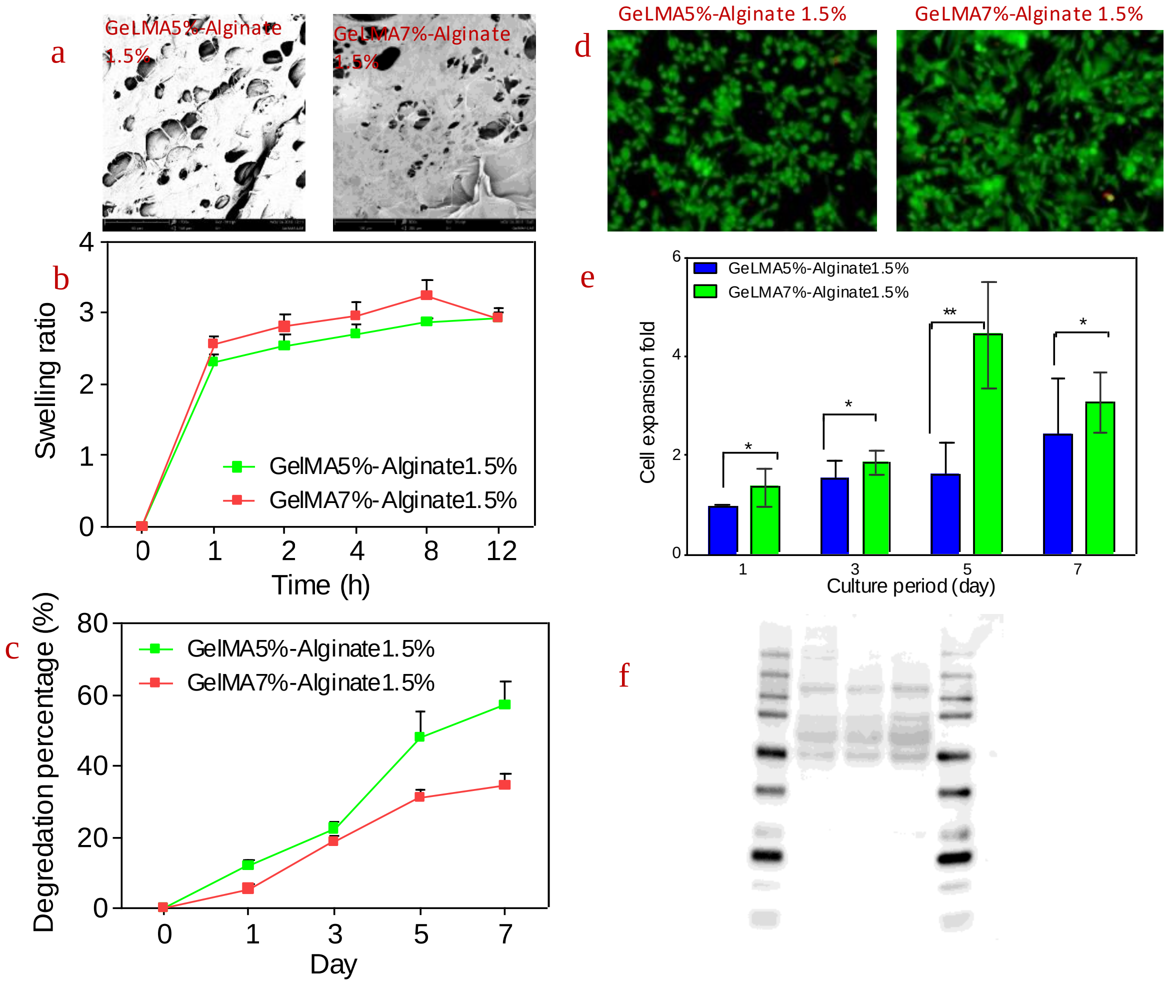

Figure S3: Evaluation of encapsulated prostate cells (PC-3) in hydrogel (a)SEM (b) swelling ratio (c) degradation percentage of various hydrogel composition (d) cell staining by live & dead assay (day 1) (e) cell expansion based on presto blue assay (f) Western Blot of encapsulated cells
